# Low testosterone and cardiometabolic risks in a real-world study of US male firefighters

**DOI:** 10.1038/s41598-021-93603-z

**Published:** 2021-07-09

**Authors:** Sushant M. Ranadive, Adriana Lofrano-Porto, Edgard M. K. V. K. Soares, Lauren Eagan, Luiz Guilherme Grossi Porto, Denise L. Smith

**Affiliations:** 1 Department of Kinesiology, School of Public Health, University of Maryland, College Park, MD USA; 2 Adrenal and Gonadal Diseases Clinic, Section of Endocrinology and Metabolism of the University Hospital, Graduate Program in Health Sciences, University of Brasilia, UnB, Brasília, Brazil; 3 Study Group on Exercise and Physical Activity Physiology and Epidemiology (GEAFS), Exercise Physiology Laboratory, Faculty of Physical Education, University of Brasilia, UnB, Brasília, Brazil; 4 First Responder Health and Safety Lab, Department of Health and Human Physiological Sciences, Skidmore College, Saratoga Springs, NY USA

**Keywords:** Endocrinology, Endocrine system and metabolic diseases

## Abstract

Low serum total testosterone (TT) is associated with increased cardiovascular risk and metabolic derangements, with fatty liver (FL) emerging as an additional cardiometabolic threat. We investigated the associations between TT and cardiometabolic (CM) health in 298 US male firefighters. Cross-sectional data from occupational health examination were analyzed. TT was categorized as low (< 264 ng/dL), borderline (264–399 ng/dL), and reference (400–916 ng/dL). Conventional CM risk factors were compared among TT categories, and between firefighters with and without FL. 81% of firefighters were obese/overweight; almost 40% had FL. In the low-TT group, only 3.1% had normal BMI, while 78.1% had FL. The low-TT group had a worse CM profile, independently of age and BMI, and a fourfold higher adjusted odds of having FL. FL was associated with lower TT, regardless of age, BMI and HbA1c. Having a FL, HbA1c ≥ 5.7% or triglycerides ≥ 150 mg/dL increased the odds for low-TT by 4.1, 2.7 and 6.6 times, respectively. These real-world data reveal strong associations between low-TT and CM risk factors and support a call for action towards screening for low-TT and FL, regardless of age, BMI or dysmetabolic conditions in firefighters. Recognizing cardiometabolic risks in firefighters provides an opportunity to lessen cardiovascular diseases burden.

## Introduction

Strenuous firefighting activity causes multiple changes in the cardiovascular system^1^, including impaired endothelial function^2^, diastolic dysfunction^2^, and an increased coagulatory state^3,4^. Sudden cardiac events are a leading cause of duty related death among firefighters, accounting for approximately 50% of line of duty deaths each year based on the National Fire Protection Association^5^. Further, firefighters are at a greater risk of sudden cardiac events during and shortly after fire suppression activities than on station duties^6,7^.

In the last decade, significant associations between endogenous total testosterone (TT) and cardiovascular (CV) health have been observed. Testosterone levels in the normal reference range have been positively associated with CV health^8,9^ in men, while low TT has been linked with higher cardiovascular morbi-mortality^10^ and multimorbidity^11^. A direct or reverse causality is yet to be clarified^12^ due to complex interactions between TT and CV health, especially in firefighters. Testosterone deficiency (TD) and CV diseases share common risk factors, such as obesity and metabolic syndrome^13,14^, which are highly prevalent among firefighters^15–18^.

Clinically, low testosterone is most commonly associated with increased visceral obesity, loss of muscle mass, fatigue, loss of bone strength, altered mood and low sex drive^8^. In regards to firefighters, the screening for testosterone deficiency is particularly relevant because: (a) firefighting independently induces a pro-coagulatory state^4,19,20^; (b) firefighting can impair vascular function which may be further potentiated by testosterone imbalances; (c) firefighters commonly exhibit multiple risk factors that are associated with low testosterone levels, including obesity, metabolic dysfunction and sleep disturbance^21–23^. However, there is limited data reporting the relationship between cardiometabolic health risks and endogenous testosterone levels among firefighters.

Interestingly, most of the preventable cardiometabolic factors are also strongly related to non-alcoholic fatty liver disease (NAFLD), a rapidly emerging inflammatory liver disease that has also been related to cardiovascular morbidity and mortality worldwide^24,25^. In this context, an independent role of NAFLD in CV health remains underexplored, despite of increasing evidence of the occurrence of common pathogenic mechanisms^24–28^. Several integrating processes linking NAFLD to the complex network of cardiometabolic factors have been proposed, including low-grade inflammation due to increased secretion of deleterious hepatokines, hyperinsulinemia and hyperglycemia, atherogenic dyslipidemia, endothelial disfunction and thrombogenesis^29^. However, to our knowledge, there is no previous study on the prevalence of NAFLD in firefighters, which is remarkable considering the high prevalence of traditional CV risk factors among these men^17,18,21,22^.

Therefore, the purpose of this study is to explore the association between endogenous serum testosterone levels and measures of cardiometabolic health, including traditional cardiometabolic risk factors and the presence of fatty liver among US firefighters in a real-world scenario. We hypothesize that firefighters presenting with lower testosterone levels have a more unfavorable overall cardiometabolic profile and higher prevalence of fatty liver disease, compared to those with testosterone levels in the reference range.

## Methods

### Study design and subjects

Using a cross-sectional study design, we evaluated occupational health records from a convenience sample of US male career firefighters who had serum testosterone levels and standard cardiovascular disease risk factors assessed as part of an occupational health evaluation. The present study is a novel research arm of a recently published study from our group. The initial study population included all male firefighters (n = 341) in a fire department in Florida who were cleared for firefighting duty and had a normal ejection fraction (> 55%), as previously described^16^. For the current study, 23 subjects were excluded due to the absence of two or more results in the studied variables (missing data), and 11 due to elevated testosterone levels at the time of the occupational health evaluation (> 916 ng/dL). Additionally, 8 subjects were excluded for reporting the use of testosterone formulations (n = 6) or of medications that induce testosterone production (clomiphene and anastrazole; n = 2). *One insulin pump user was excluded due to acute diabetic decompensation at the time of the evaluation. None of the firefighters used medications that could impact the risk of fatty liver disease, such as amiodarone, methotrexate, glucocorticoids, valproate and anti-retrovirals, among others.* Thus, our final sample consisted of 298 male career firefighters.

The occupational medical evaluation was required of all members based on fire department policies and was provided by a contractual arrangement with an occupational health clinic. Components of the medical evaluation included a physical examination, collection of anthropometric data, cardiometabolic blood chemistry panel, ultrasonographic screening of fatty liver, and serum total testosterone level (TT).

Data were examined retrospectively. The occupational health clinic sent deidentified medical records to the research team who extracted relevant data. The study was performed in accordance with the Declaration of Helsinki and all methods were performed in accordance with the relevant guidelines and regulations. Informed consent was waived by the Skidmore College Institutional Review Board since researchers received deidentified data and due to the retrospective nature of the study. The study protocol was approved by the Skidmore College Institutional Review Board.

### Data assessment and analysis

Height and weight were assessed using a stadiometer and physician scale. BMIs were calculated as weight (kg)/height squared (m^2^). Resting blood pressure (BP) was measured by a trained health care provider with the firefighter in a seated position^30^.

Blood samples from eight to ten hours fasting were obtained in the early morning hours and were analyzed for blood biochemistry, which included glucose, HbA1c, lipid profile and basic liver function tests (AST, ALT) as primary variables. Serum total testosterone (TT) was assessed using a standard electrochemiluminescence assay (ECLIA, Roche Diagnostics, Indianapolis, IN, USA). Reference interval for adult males was determined by the clinical laboratory based on standardization of the assay to the CDC reference method (labcorp.com/assets/11476). All blood analysis were performed by LabCorp (Laboratory Corporation of America, Burlington, NC, USA).

Serum TT values were categorized using the standard adult male reference range based on the Center of Disease Control (CDC, USA) reference method, as recommended by the Endocrine Society (264 to 916 ng/dL)^8,31^, resulting in the following groups: low (< 264 ng/dL), borderline (264–399 ng/dL), reference (400–916 ng/dL) and high testosterone (> 916 ng/dL). Due to the possibility of unreported exogenous testosterone supplementation or over-the-counter testosterone stimulants, the high testosterone group was not analyzed and therefore excluded from the study in order to avoid misinterpretation or artificial overestimation of associations among the variables.

Liver ultrasonography was performed by trained technicians at the occupational medicine clinic as part of an abdominal scan using a conventional B-mode ultrasound. Parenchymal echotexture was routinely assessed to estimate the degree of diffuse fatty infiltration in the liver. Findings consistent with fatty liver include hyperechoic parenchymal texture and liver brightness, contrast between the liver and the kidney, and impaired appearance of the intrahepatic vessels and diaphragm^32,33^.

### Statistical analyses

The normality of the variables’ distributions was confirmed in the groups and subgroups using the Kolmogorov–Smirnov test. Variables that were possibly not normally distributed were further examined using the Q-Q Plot^34^ and normality was confirmed. Homogeneity of variance was also confirmed using Levene’s test in all groups and subgroups. Descriptive statistics for serum total testosterone and cardiometabolic health parameters are presented as continuous and categorical variables. To compare the effect of different testosterone levels on cardiometabolic variables an ANOVA was performed using Tukey's post hoc analysis. We also performed a T-test comparison between those with increased (≥ 5.7%) *vs* normal HbA1c (< 5.7%)^35^, those with the ultrasonographic-defined fatty liver *vs* those without it, those with high triglycerides (≥ 150 mg/dL) *vs* normal triglycerides (< 150 mg/dL), and firefighters with low (< 40 mg/dL) *vs* normal HDL-cholesterol (HDL) (≥ 40 mg/dL)^36^. Chi-squared test was used to analyze the association between TT and positive fatty liver and abnormal categories of HbA1c, triglycerides and HDL. To express the strength of association, the crude and the adjusted odds ratios (OR) and 95% confidence intervals (95% CI) were calculated. IBM SPSS Statistics^®^ 21 (IBM Corporation, USA) software package was used for data processing and analysis and Prism 8 for Windows (GraphPad Software, USA) for graphic design. The differences were considered statistically significant when a two-tailed P-value was less than or equal to 5% (≤ 0.05).

## Results

This is a cohort of young and middle-aged firefighters, ranging from 19 to 60 years old. Almost 85% of the participants were 50 or less, and 99.7% were below 60 years old. The mean BMI was in the overweight range, varying from 19.1 to 45.5 kg/m^2^. Despite the relatively low average age (37.6 ± 10.2 years old), only 18.8% of the sample (n = 56) had normal BMI, while 45.3% (n = 135) were overweight, and 35.9% (n = 107) were in the obese range; 35.6% (n = 106) had abnormal resting blood pressure (≥ 140 mmHg systolic and/or ≥ 90 mmHg diastolic), and 13.4% (n = 40) had HbA1c levels at the diabetic or pre-diabetic ranges (≥ 5.7%). Of all firefighters included in the study, 12.4% (n = 37) have been treated with at least one anti-hypertensive, 5.0% (n = 15) with lipid-lowering agents, 1.3% (n = 4) with anti-diabetics, and in 1.7% (n = 5) of the participants data on medication use was not available for the study. Mean TT value was in the reference range but there was a high interindividual variation. Characteristics of the study population are presented in Table 1.

**Table 1:** Characteristics of the study population.

	Participants (n = 298)
Serum total testosterone (ng/dL)	452.7 ± 163.0
Age (yrs)	37.6 ± 10.2
BMI (kg/m^2^)	28.9 ± 4.5
Systolic BP (mmHg)	121.4 ± 7.6
Diastolic BP (mmHg)	77.2 ± 5.5
HbA1c (%)	5.4 ± 0.7
Glucose (mg/dL) ^a^	92.6 ± 14.3
TC (mg/dL)	185.5 ± 37.9
HDL (mg/dL)	48.6 ± 11.6
LDL (mg/dL)^b^	111.4 ± 32.7
Triglycerides (mg/dL)	130.5 ± 86.3
AST (IU/L)	24.2 ± 8.4
ALT (IU/L)	30.1 ± 16.4

Variables are expressed as mean ± standard deviation; BMI: body mass index; BP: blood pressure; HbA1c: glycated hemoglobin; Different sample sizes for the variables due to missing data: ^a^Sample size is n = 293; ^b^Sample size is n = 294.

**Figure 1:**
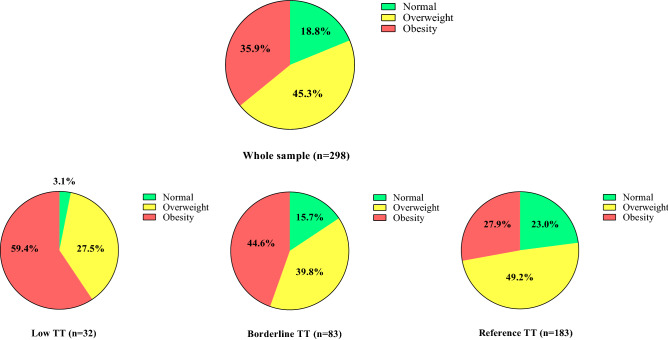
Distribution of firefighters of different BMI categories among total testosterone groups, Low TT (< 264 ng/dL), Borderline TT (264–399 ng/dL), and Reference TT (400–916 ng/dL).

A descriptive analysis of BMI among all men separated by TT groups is presented in Fig. 1. In the low TT group, only 3.1% of the firefighters had BMI in the normal range.

Comparisons of cardiometabolic variables based on TT groups are shown in Table 2. Of note, the low-TT group was older, and had higher BMI. Systolic and diastolic blood pressure were also significantly higher in the low testosterone group, though still with mean values in the normal range.

**Table 2:** Comparison of cardiometabolic parameters among firefighters within different categories of serum total testosterone levels.

	Low TT (< 264 ng/dL) n = 32	Borderline TT (264–399 ng/dL) n = 83	Reference range TT (400–916 ng/dL) n = 183	p-value
Serum total testosterone (ng/dL)	210.6 ± 51.6^a,b^	330.0 ± 40.7^a^	550.6 ± 124.0	< 0.01
Age (years)	42.5 ± 9.3^a^	37.9 ± 10.7	36.6 ± 9.9	0.01
BMI (kg/m^2^)	32.4 ± 5.4^a,b^	29.7 ± 4.1^a^	28.0 ± 4.1	< 0.01
Systolic BP (mmHg)	125.6 ± 6.5^a^	122.9 ± 8.2^a^	120.0 ± 7.0	< 0.01
Diastolic BP (mmHg)	79.2 ± 4.0^a^	77.9 ± 5.5	76.5 ± 5.6	0.01
HbA1c (%)	5.6 ± 0.7	5.5 ± 0.6	5.3 ± 0.7	0.14
Glucose (mg/dL)*	94.1 ± 14.3	93.6 ± 13.2	91.9 ± 14.3	0.54
TC (mg/dL)	196.1 ± 43.0	184.4 ± 40.1	184.2 ± 35.8	0.25
HDL (mg/dL)	42.7 ± 9.0^a^	45.0 ± 9.8^a^	51.3 ± 11.9	< 0.01
LDL (mg/dL)^#^	116.4 ± 38.8	111.1 ± 36.9	110.7 ± 29.5	0.67
LDL/HDL^#^	2.77 ± 0.95^a^	2.54 ± 1.00^a^	2.27 ± 0.75	< 0.01
VLDL (mg/dL)^#^	36.1 ± 15.6^a,b^	28.0 ± 14.0^a^	22.2 ± 13.8	< 0.01
N-HDL cholesterol (mg/dL)	153.4 ± 41.3^a^	139.4 ± 41.0	133.0 ± 35.6	0.02
Triglycerides (mg/dL)	188.8 ± 89.5^a,b^	147.6 ± 97.7^a^	112.5 ± 73.8	< 0.01
AST (IU/L)	26.8 ± 9.5	23.4 ± 8.3	24.2 ± 8.2	0.15
ALT (IU/L)	40.7 ± 15.6^a,b^	30.8 ± 19.2	28.0 ± 14.5	< 0.01
AST/ALT	0.69 ± 0.19^a,b^	0.86 ± 0.30^a^	0.95 ± 0.28	< 0.01

^a^Significantly different when compared to reference group (Tukey’s post-hoc); ^b^Significantly different when compared to borderline group; Different sample sizes for the variables: *Low TT (n = 31), Borderline TT(n = 81), and Reference TT (n = 181); ^**#**^Low TT(n = 31), Borderline TT(n = 81), and Reference TT (n = 182). BMI (Body Mass Index); BP: Blood Pressure; HbA1c: Glycated Hemoglobin; TC: Total cholesterol; HDL: High Density Lipoprotein-cholesterol; LDL: Low Density Lipoprotein-cholesterol; VLDL: Very Low Density Lipoprotein- cholesterol; AST: Aspartate Aminotransferase; ALT: Alanine Aminotransferase.

**Figure 2:**
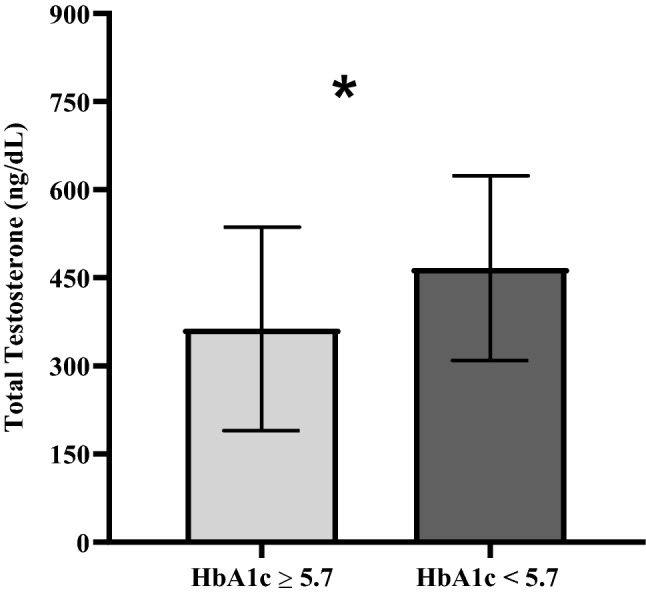
Serum Total testosterone levels (Mean ± SD) in firefighters with increased (n = 40) vs normal Hb1Ac (n = 258); *(p < 0.05; T-test).

Laboratory parameters commonly used to assess cardiometabolic risk in clinical practice significantly differed among TT groups. Most importantly, significant differences in the blood lipid profile were observed, including a lower HDL and higher triglycerides in both low and borderline TT groups compared to firefighters with TT in the reference range. Consistently, non-HDL cholesterol levels were also higher in the low TT group. Fasting glucose and HbA1c did not differ among TT categories (Table 2). However, serum TT values were significantly lower in firefighters with HbA1c levels in the pre-diabetic or diabetic range (≥ 5.7%) compared to those with normal HbA1c (Fig. 2). Remarkably, ALT levels were significantly higher in the low TT group, thus revealing a lower AST/ALT ratio in this group (Table 2).

**Figure 3:**
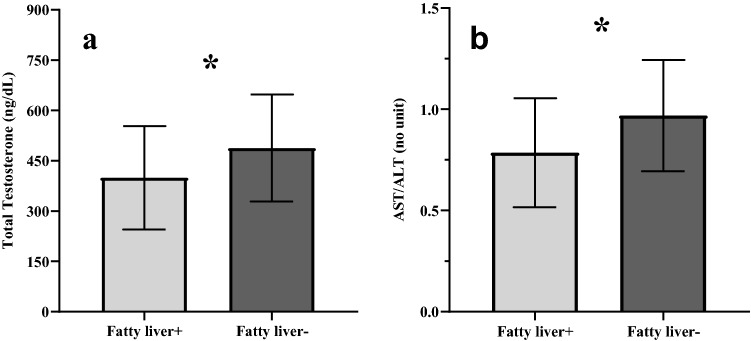
Serum total testosterone levels (**a**) and AST/ALT ratio (**b**) (mean ± SD) in firefighters with fatty liver (fatty liver + [n = 119]) and in those without it (fatty liver− [n = 179]). *p < 0.01 (T-test).

The overall prevalence of fatty liver identified by ultrasound (US) in our cohort is approximately 40% (119/298), and 78.1% among firefighters with low TT (25/32). We found significant associations between the presence of fatty liver and conventional cardiometabolic risk factors (glucose, HbA1c and lipid profile), as well as with testosterone levels. Firefighters with fatty liver showed significant unfavorable differences in most of the cardiometabolic variables as compared to those without fatty liver (Table 3). Although in the upper limit of normality or only slightly increased, the statistically significantly higher ALT levels in the group with fatty liver are noteworthy. The fatty liver group also showed significantly lower testosterone levels as well as lower AST/ALT ratios (Fig. 3a,b).

**Table 3:** Comparison of primary cardiometabolic parameters among firefighters with (fatty liver +) and without fatty liver on ultrasound (fatty liver −).

	Fatty liver + (n = 119)	Fatty liver − (n = 179)	p-value
Serum total testosterone (ng/dL)	399.3 ± 154.1	488.2 ± 159.5	< 0.01
Age (years)	42.2 ± 9.3	34.5 ± 9.6	< 0.01
BMI (kg/m^2^)	31.5 ± 4.6	27.3 ± 3.5	< 0.01
Systolic BP (mmHg)	124.0 ± 7.1	119.7 ± 7.4	< 0.01
Diastolic BP (mmHg)	78.4 ± 5.4	76.3 ± 5.3	< 0.01
Glucose (mg/dL)^a^	96.8 ± 19.5	89.8 ± 8.5	< 0.01
HbA1c (%)	5.6 ± 1.0	5.3 ± 0.4	< 0.01
TC (mg/dL)	193.6 ± 38.4	180.1 ± 36.6	< 0.01
HDL (mg/dL)	45.5 ± 11.1	50.7 ± 11.4	< 0.01
LDL^b^ (mg/dL)	117.4 ± 34.3	107.5 ± 31.1	0.01
LDL/HDL^b^	2.68 ± 0.92	2.22 ± 0.78	< 0.01
VLDL (mg/dL)^b^	30.3 ± 14.2	22.0 ± 14.2	< 0.01
N-HDL cholesterol (mg/dL)	148.3 ± 37.7	129.4 ± 36.7	< 0.01
Triglyceride (mg/dL)	158.3 ± 89.3	112.0 ± 79.3	< 0.01
AST (IU/L)	25.0 ± 8.8	23.7 ± 8.1	0.21
ALT (IU/L)	35.8 ± 20.2	26.4 ± 12.1	< 0.01
AST/ALT	0.79 ± 0.27	0.97 ± 0.27	< 0.01

Different sample size for the variables due to missing data: ^a^Fatty liver+ (n = 116), Fatty liver− (n = 177); ^b^fatty liver+ (n = 116), Fatty liver− (n = 178).

A similar pattern of unfavorable associations as seen when comparing those with and without fatty liver for all cardiometabolic variables when groups were categorized by clinically relevant values: HbA1c (≥ 5.7 *vs.* < 5.7%), triglycerides (≥ 150 *vs.* < 150 mg/dL), and HDL (< 40 *vs.* ≥ 40 mg/dL). Remarkably, TT levels were also significantly lower in each metabolically abnormal categories (p < 0.01) (data not shown).

After adjusting for confounding variables, the odds of having fatty liver was almost fourfold higher for the low-TT group as compared to the reference TT group (Table 4). The reverse analysis also showed a strong association between TT and fatty liver. Those with fatty liver have an adjusted odds-ratio of 4.1 (95%CI 1.5–11.3) of having low TT as compared to those without.

**Table 4:** Odds ratio of having various metabolic abnormalities in male career firefighters with borderline or low total testosterone (TT) levels compared to those with TT in the reference range.

Total testosterone group	n	%	Crude analysis		Adjusted analysis^*#*^	
			OR (95%CI)	p	OR (95%CI)	p
**Fatty liver + **						
Reference range TT (400–916 ng/dL)	183	31.1	1.00	–	1.00	–
Borderline TT (264–399 ng/dL)	83	44.6	1.78* (1.04–3.03)	0.04	1.29 (0.69–2.41)	0.43
Low TT (< 264 ng/dL)	32	78.1	7.90* (3.23–19.31)	< 0.01	3.99* (1.45–10.94)	0.01
**HbA1c ≥ 5.7%**						
Reference range TT (400–916 ng/dL)	183	8.2	1.00	–	1.00	–
Borderline TT (264–399 ng/dL)	83	16.9	2.27* (1.04–4.96)	0.04	1.79 (0.78–4.10)	0.17
Low TT (< 264 ng/dL)	32	34.4	5.87* (2.38–14.44)	< 0.01	2.76* (1.01–7.53)	0.05
**Triglycerides (≥ 150 mg/dL)**						
Reference range TT (400–916 ng/dL)	183	18.6	1.00	–	1.00	–
Borderline TT (264–399 ng/dL)	83	33.7	2.23* (1.24–4.02)	0.01	1.86* (1.00–3.46)	0.05
Low TT (< 264 ng/dL)	32	68.8	9.64* (4.18–22.23)	< 0.01	6.36* (2.62–15.37)	< 0.01
**HDL-cholesterol < 40 mg/dL**						
Reference range TT (400–916 ng/dL)	183	12.6	1.00	–	1.00	–
Borderline TT (264–399 ng/dL)	83	27.7	2.67* (1.39–5.10)	< 0.01	2.34* (1.20–4.57)	0.1
Low TT (< 264 ng/dL)	32	37.5	4.17* (1.80–9.66)	< 0.01	2.42 (0.94–6.28)	0.07

TT: total testosterone; p: Pearson’s Chi-square p-value.

^#^Adjusted for body mass index, age, and HbA1c; *Significantly different when compared to reference group.

When categorizing the groups by HbA1c and triglycerides, the adjusted odds of having altered HbA1c (≥ 5.7%) or high triglycerides (≥ 150 mg/dL) were 2.8 (1.0–7.5) and 6.4 (2.6–15.3), respectively, among firefighters in the low-TT group as compared to the reference TT group (Table 4).

## Discussion

To the best of our knowledge, this is the first study to report the relationship between serum testosterone levels with conventional cardiometabolic variables and fatty liver in the US fire service, an important public safety group that has an unacceptably high rate of duty-related sudden cardiac deaths. Our main findings among young/middle aged career firefighters were that : (a) firefighters in the low-TT group have a worse overall cardiovascular risk profile, including the presence of fatty liver on ultrasound, independently of age and BMI; (b) the prevalence of fatty liver identified by ultrasound in the whole cohort is high (± 40%), and even higher among those men with low TT (78.1%); (c) fatty liver was associated with lower TT levels regardless of age, BMI and HbA1c, and other cardiometabolic risk factors; and (d) having a fatty liver, altered HbA1c or high triglycerides increases the odds for presenting with low TT levels by 4.1, 2.7 and 6.6 times, respectively.

The associations of TT levels with age and BMI are well established and support our multivariate approach. The prevalence of testosterone deficiency (TD) is positively and linearly correlated with age in the general population as well as among military personnel^8,37,38^. Among obese men, it has been shown to be as high as 78%^39^. We found a prevalence of 38.6% of low or borderline TT levels in this relatively young cohort of firefighters raising important concerns that might be specific for firefighter’s health. Perhaps even more concerning, we found increased risk (high odds ratio) of having low TT among young/middle-aged firefighters with fatty liver or abnormal glucose and lipid profiles, even when controlling for common confounders.

Our real-world data from annual occupational medical exams extend previous research by presenting novel findings about the association of low testosterone levels and cardiometabolic health in men, including traditional risk factors and fatty liver—an emerging cardiometabolic threat. The high prevalence of ultrasonography defined fatty liver and its association with low testosterone levels among firefighters represents a novel and relevant finding, beyond its expected association with conventional cardiometabolic risk factors. While the estimated prevalence of non-alcoholic fatty liver in the US general population, also identified by ultrasound, is around 24%^40^, we found that 25 out of 32 middle-aged firefighters with low TT had fatty liver (78%). Further, the prevalence of 53.9% of fatty liver among firefighters with TT below the reference range (≤ 400 ng/dL) is remarkable.

The fact that approximately half of duty-related deaths among US firefighters are due to sudden cardiac events lends urgency to unravel the complex cardiometabolic interactions that drive the risk of major adverse cardiovascular events in these men. The odds for severe cardiovascular events among firefighters are not random, but are associated with physically and psychologically stressful job tasks^6,7,41^. Firefighters’ duty-related deaths attributed to coronary heart disease and increased heart size were associated with a stunning 112-fold increased risk of cardiac death during fire suppression activities compared with non-emergency duties at the fire station^6^. Thus, a major challenge for the fire service and for the occupational health team is the early identification of the most susceptible workers^42^, and strategies and policies to prevent the advancement of cardiovascular disease. Recently, a study in a German Fire Service reported that firefighters with higher cardiorespiratory fitness levels had more favorable cardiovascular risk parameters, namely lower BMI, waist circumference, body fat, resting systolic blood pressure and triglycerides^43^.

Two recent literature reviews have pointed out for an important association between fatty liver disease and CV events, and its possible independent effect on cardiac risk independent of traditional CV risk factors, such as hypertension and dyslipidemia^26,27^. NAFLD was associated with an increased incidence of CV disease in DM2 patients, independent of traditional CV risk factors and metabolic syndrome components, i.e., atherogenic dyslipidemia, dysglycemia, obesity and hypertension^44^. In addition, it has been proposed that the concomitance of dyslipidemia and NAFLD in a single individual could enhance atherogenic activity, especially in the presence of high LDL/VLDL, low HDL, and high triglycerides^27^. Although our study was not designed to address CV outcomes, most of our fatty liver cases are associated with at least one of the dysmetabolic conditions that frequently affect firefighters and increase their overall CV risk. Interestingly, the mean lipid profile in our cohort is only mildly abnormal, which could be partially related to the use of lipid-lowering drugs, relatively young age of our sample or behavioral or environmental factors that were not addressed in the study. Nevertheless, the major lipid alterations were found in the low-TT and fatty liver positive groups, including low HDL-cholesterol and high triglycerides and non-HDL cholesterol (Tables 1, 2, 3).

Previous research have shown systemic microvascular damage associated with endothelial dysfunction related to persistent inflammation, subclinical atherosclerosis, structural cardiac abnormalities, and oxidative stress, all possible mechanisms underlying the increased CV risk associated with NAFLD^26,27,45,46^. These findings are of particular concern among firefighters due to the acute effects of firefighting on vascular function^2,20^ and the high prevalence of left ventricular hypertrophy or cardiomegaly in autopsies of firefighters who died due to cardiac conditions^47^. Of note, we have recently shown that firefighters with borderline-low TT levels (between 264 and 320 ng/dL) have a fourfold higher risk of having a lower left ventricular wall thickness, independently of age, BMI, and HbA1^16^. Whether the available cardiovascular findings correlate with fatty liver and/or an unfavorable overall cardiometabolic profile related to low testosterone levels is still hypothetical, thus highlighting the need for additional studies.

The present data corroborate the association of low testosterone with multiple cardiometabolic risk factors^48,49^ that have been described in the context of the fire service^22,50^. However, the design of our study does not allow us to establish causality nor exclude reverse causality between low TT and cardiometabolic risk factors, including fatty liver. On the other hand, the consistency of the differences between groups, either when categorized by TT level, presence or not of fatty liver, or by HbA1c, triglycerides and HDL-cholesterol conventional cut-off points corroborates the complex interactions between testosterone secretion and cardiovascular risk factors. The fourfold increased risk of having low testosterone among firefighters with fatty liver, and *vice-versa*, after adjustment for obesity, also strongly support the idea that fatty liver disease negatively influences circulating testosterone levels bidirectionally.

Fatty liver is linked to obesity and it appears that the combination of these two conditions is related to low testosterone in a significant percentage of men, partly due to decreased SHBG levels that frequently accompanies obesity and NAFLD^12^. However, the levels of SHBG secreted by the liver, and thus TT levels are not solely dependent of the degree of fat. For example, there is a significant role of SHBG gene polymorphisms on the levels of SHBG independently of obesity, fatty liver and testosterone. Also, the degree of inflammation, distribution of adiposity and the energetic efficiency of the different adipose tissue deposits have been studied as determinants of both NAFLD and testosterone insufficiency, beyond obesity per se^29^.

Consistent with the hypothesis of an effect of testosterone on liver fat deposition, independent of obesity, an increased fat deposition was observed in testicular feminizing mice (tfm) fed with a high-cholesterol diet, compared with their wild-type littermates. Those mice have a non-functional androgen receptor (AR) and severe testosterone deficiency. Testosterone supplementation in those animals reduced hepatic steatosis, suggesting an AR independent role on key regulatory lipogenic enzymes^51^. Another study using a liver-specific AR knock-out mouse model showed that a high-fat diet induced insulin resistance and hepatic steatosis only in the males, not in females^52^.

Furthermore, several studies have investigated cardiometabolic risks in Klinefelter syndrome. Men with this chromosomal disorder have variable degrees of testosterone insufficiency lifelong, and present an increased prevalence of metabolic syndrome, high triglycerides, type 2 diabetes mellitus and increased platelet activity, along with increased CV risk^53^. However, it is still unclear whether an unfavorable body composition and dysmetabolism in those men is a consequence of a peculiar gene expression pattern, testosterone deficiency or both. This is an especially critical clinical question because testosterone replacement only partly reverts this metabolic scenario though without definite evidence of beneficial effects on cardiovascular outcomes^54^. Overall, current knowledge points to a multifactorial interactive network, in which genetics, environmental and job-related factors, sleep pattern, diet, exercise and tissue specific testosterone-related metabolic effects, all play determinant roles on a man’s risk of having a major cardiovascular acute event^12,54^.

Apart from the strengths of our data, some limitations inherent to the study design should be pointed out. The retrospective design based on clinical charts review is subject to occasional missing data and precludes a more detailed analysis. The data from the current study does not allow for a definitive clinical diagnosis of NAFLD, since exclusion of excessive alcohol intake was not possible. On the other hand, although restricted to ultrasound-based liver appearance to screen for hepatic fatty infiltration, this is the dominant screening tool for NAFLD worldwide, due to its low cost, safety and accessibility^32^. Furthermore, although insufficient for a diagnosis of NAFLD, the higher ALT levels and, consequently, lower AST/ALT ratio in the firefighters with fatty liver corroborate previous studies^29,32^. A prospective study of 400 US military personnel and their relatives (mean age 55 years) estimated the prevalence of NAFLD by ultrasound, further confirmed by liver biopsy, to be as high as 46%. In this study, factors associated with NAFLD included male sex, increasing age, and the presence of systemic hypertension, obesity, or diabetes, similar to the findings of the current study^55^.

Lastly, it is important to point out that our study was based on a single serum TT measure, instead of at least two independent measures recommended for the clinical diagnosis of hypogonadism. TT levels are subjected to variations related to the circulating levels of Sex-Hormone Binding Globulin (SHBG), which is produced in the liver and can be mildly reduced under states of obesity, insulin resistance, and NAFLD. Also, medications routinely used by the participants, such as antihypertensive drugs (12.4%), lipid-lowering agents (5.0%) and antidiabetics (1.3%), could potentially influence the results of the cardiometabolic variables. However, we note that a relatively small proportion of participants reported taking those drugs, and further, their prescription supposes the diagnosis of hypertension, dyslipidemia and diabetes, which are considered cardiometabolic disease equivalents and thus would likely underestimate our results, rather than artificially overestimate them. Additional studies, with longitudinal designs, are necessary to confirm and clarify the direction of the relationships between endogenous low TT, fatty liver and NAFLD, and other cardiometabolic impairments in men in the general population as well as in firefighters. Importantly, our findings highlight the need to investigate the roles of testosterone replacement on cardiovascular health of men with unambiguous evidence of testosterone deficiency and NAFLD^25^.

In summary, these real-world data reinforce and extend our previous exploratory findings regarding the association between TT and cardiovascular health among firefighters, highlighting the importance of assessing serum testosterone levels in the fire service workforce, even at a relatively young age. By both an inferential or associative statistical approach, our results support two possible general interpretations: (1) that low TT levels, an unfavorable cardiometabolic profile, and the presence of fatty liver constitute “pillars” of a unique and complex metabolic clinical condition, potentially reversed if recognized early, that increases the risk of a major cardiovascular acute events; (2) that all of those factors consist of independent pathophysiological entities, that interact and aggregate cardiovascular risk one to the other. Either possibility warrants a call for action towards early screening for low TT^16^ and fatty liver^27^, regardless of age, BMI or glucose control in this unique workforce that has a higher overall CV risk due to its inherent job-related tasks^1,6^. Furthermore, unravelling cardiometabolic triggers in firefighters provides important information for the improvement of actions to prevent the growth of cardiovascular diseases globally.

## Conclusions

Our data confirm the preliminary hypotheses that firefighters in the low endogenous serum testosterone group had a more unfavorable cardiometabolic profile compared to those in the testosterone reference range, including a higher prevalence of fatty liver. A high overall prevalence of ultrasonographic-defined fatty liver was observed in this population, that was even more pronounced in men in the low TT group. The odds of having fatty liver, abnormal glucose and lipid profiles were significantly higher among firefighters with low TT as compared to those in the reference range of TT. In this young and middle-aged career cohort of firefighters we found significant associations between low serum testosterone level, a worse traditional CV risk profile and the presence of fatty liver. Those in the low-TT group were older, had higher BMI, a higher Hba1c and had a more impaired lipid profile. Importantly, the presence of fatty liver was associated with lower testosterone levels regardless of age, BMI and HbA1c.

Our results provide a starting rationale for early screening of both testosterone deficiency and fatty liver in firefighters along with other CV risk factors. This would allow evidence-based clinical follow up and referral for those who might benefit from timely and individualized pharmacological and/or non-pharmacological therapies, thus avoiding expensive high complexity procedures, and promoting a better quality of life. Our data extend previous literature findings^56,57^ that support early and continuous implementation of healthy lifestyle strategies based on physical activity/fitness promotion and good quality diets as well^58^.
