# Author Correction: Low testosterone and cardiometabolic risks in a real-world study of US male firefighters

**DOI:** 10.1038/s41598-022-10469-5

**Published:** 2022-04-12

**Authors:** Sushant M. Ranadive, Adriana Lofrano‑Porto, Edgard M. K. V. K. Soares, Lauren Eagan, Luiz Guilherme Grossi Porto, Denise L. Smith

**Affiliations:** 1 Department of Kinesiology, School of Public Health, University of Maryland, College Park, MD USA; 2 Adrenal and Gonadal Diseases Clinic, Section of Endocrinology and Metabolism of the University Hospital, Graduate Program in Health Sciences, University of Brasilia, UnB, Brasília, Brazil; 3 Study Group on Exercise and Physical Activity Physiology and Epidemiology (GEAFS), Exercise Physiology Laboratory, Faculty of Physical Education, University of Brasilia, UnB, Brasília, Brazil; 4 First Responder Health and Safety Lab, Department of Health and Human Physiological Sciences, Skidmore College, Saratoga Springs, NY USA

Correction to: *Scientific Reports* 10.1038/s41598-021-93603-z, published online 09 July 2021

The Funding section in the original version of this Article was omitted. The Funding section now reads:

“This publication was made possible by Federal Emergency Management Agency (FEMA), Grant/Award Number: EMW – 2017 – FP – 00445 (author DLS), Fundação de Apoio à Pesquisa do Distrito Federal, Grant/Award Number: 3/2016 (author LGGP) and Coordenação de Aperfeiçoamento de Pessoal de Nível Superior - Finance Code 001: EMKVKS was supported by a CAPES scholarship (author EMKVKS).”

The original Article has been corrected.

